# Peri‐implant soft tissue contour after stepwise replacement of missing and ankylotic central maxillary incisors in young adult: A clinical case report

**DOI:** 10.1002/ccr3.4960

**Published:** 2021-10-13

**Authors:** Rok Gašperšič, Alja Cmok Kučič, Karmen Volk Gašperšič, Rok Kosem

**Affiliations:** ^1^ Department of Periodontology, Faculty of Medicine University of Ljubljana Ljubljana Slovenia; ^2^ Public Health Center Celje Celje Slovenia; ^3^ Private Practice Ljubljana Slovenia; ^4^ Department of Paediatric Dentistry University Medical Centre Ljubljana Ljubljana Slovenia

**Keywords:** ankylosis, avulsion, connective tissue graft(s), guided bone regeneration, implantology, ridge augmentation, ridge preservation

## Abstract

Replantation and retention of ankylosed tooth after pubertal growth spurt enables stepwise replacement of both central incisors with implants. Partial extraction contributes to natural gingival contour.

## Graphical abstract

Replantation and retention of ankylosed tooth after pubertal growth spurt enable stepwise replacement of both central incisors with implants. Partial extraction contributes to natural gingival contour.

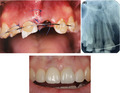

## 1 INTRODUCTION

A 13‐year‐old girl experienced avulsion of both maxillary central incisors with only one (#11) being replanted. Through a decade lasting carefully planned conservative, surgical and restorative stepwise approach, temporary retention and partial extraction of ankylosed tooth enabled preservation of tissue architecture and successful rehabilitation with two implants.

Avulsion of permanent teeth is one of the most serious dental injuries and one of a few real emergencies in dentistry usually happening in childhood or adolescence.^1^ Tooth avulsion implies total displacement of the tooth out of its socket.^2^ Various statistics have shown that avulsion of permanent teeth represents 0.5–3% of all dental injuries.^1^ Most frequently affected teeth are maxillary central incisors. Avulsion usually involves a single tooth and multiple avulsions are only occasionally encountered.^2^ Long‐term prognosis of such teeth and periodontal tissues depends on emergency actions taken at the time of accident and immediately after avulsion. A critical decision for replantation of avulsed tooth after extended extra‐alveolar time leads to many biological considerations and questionable long‐term outcomes, in particular, if ankylosis follows as a result of unsuccessful periodontal regeneration.^1^

Negative aesthetic sequels of severe dental trauma in children and young adults that result in the loss of affected teeth or their ankylosis include not only vertical tissue defect due to the arrest in alveolar growth, alveolar ridge resorption, collapse of the gingival architecture, tilting, and infraocclusion of adjacent teeth but also psychological distress. When multiple teeth in the aesthetic region are lost or ankylosed, disturbances in alveolar process growth are even more pronounced.^2^ When implant‐supported rehabilitation is considered, additional problems include an imperative for postponing dental implant insertion by the end of skeletal growth^3^ and difficulties to reconstruct gingival architecture including inter‐implant papillae when placing adjacent implants in the aesthetic zone.^4^, ^5^

The main purpose of treatment in the reported case was to overcome such challenges with combined efforts and skills of an interdisciplinary team, representing pediatric dentist, periodontist and restorative dentist, working on a pre‐determined stepwise treatment plan in order to re‐create the aesthetic appearance and function of dentition after avulsion that resulted in loss of two central maxillary incisors in a young adult. The presence of an ankylotic tooth which was not causing serious complications was exploited to preserve tissue architecture. With a temporarily preserved ankylotic incisor, the first implant was inserted with a simultaneous guided bone regeneration procedure. In the second stage when the second incisor was removed, the modified socket‐shield technique was used to preserve the marginal tissue architecture at the second implant site. The objective of our treatment was to provide the patient with acceptable provisional prosthesis until the end of skeletal growth and to exploit all the possibilities of one ankylosed incisor to achieve aesthetically acceptable outcome after bone and soft tissue regeneration and rehabilitation with two implant‐supported crowns.

## 2 CLINICAL CASE

### 2.1 Case presentation

**FIGURE 1:**
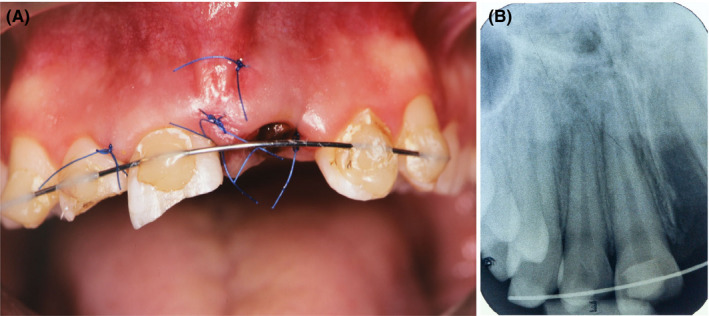
Clinical condition 7 days after tooth #11 replantation and immobilization (A). Radiographically appropriate position of tooth #11 after replantation with empty alveolar socket in the region of #21 (B)

A 13‐year‐old girl with adolescence‐associated delicate psychological behavior and a gummy smile suffered dental trauma with avulsion of both upper central incisors after collision with another person while water sliding. After 30 min search in the pool water, only tooth #11 was found and held intraorally in the patient's saliva. First aid was provided to the patient 3 h later by a maxillofacial surgeon and included delayed replantation with rigid splinting of tooth #11. Tooth #21 was never found. Initial examination at Dental Clinic, UMC Ljubljana, 7 days after trauma demonstrated normal pulp sensitivity test results for adjacent teeth #13, #12, #22, #23 with no subjective nor clinical signs of inflammation. A comprehensive evaluation indicated that the collision resulted in avulsion of teeth #11, #21, subluxation of #12, and uncomplicated enamel‐dentin crown fracture of #11 and #12 (Figure 1A).^6^ Tooth #11 had radiographically appropriate intraalveolar position with negative pulp sensibility response. After removal of rigid splint, tooth #11 and tooth #12 showed increased mobility and both experienced sensitivity upon percussion (Figure 1B). Besides having asthma, the patient was in good general health without medication on a regular basis.

### 2.2 Case management

Several surgical and prosthetic factors were assessed when replacement of maxillary central incisors with implants turned into a final strategic treatment plan. Soft tissue contour with inter‐implant papillary tissue configuration became a parameter that was difficult to obtain and required precise planning of conservative, surgical and restorative steps. All critical points and treatment alternatives were explained in detail to the patient and her parents. Consent for treatment was obtained from parents when the patient was under‐aged and from the patient before each surgical procedure.

#### 2.2.1 Conservative treatment

**FIGURE 2:**
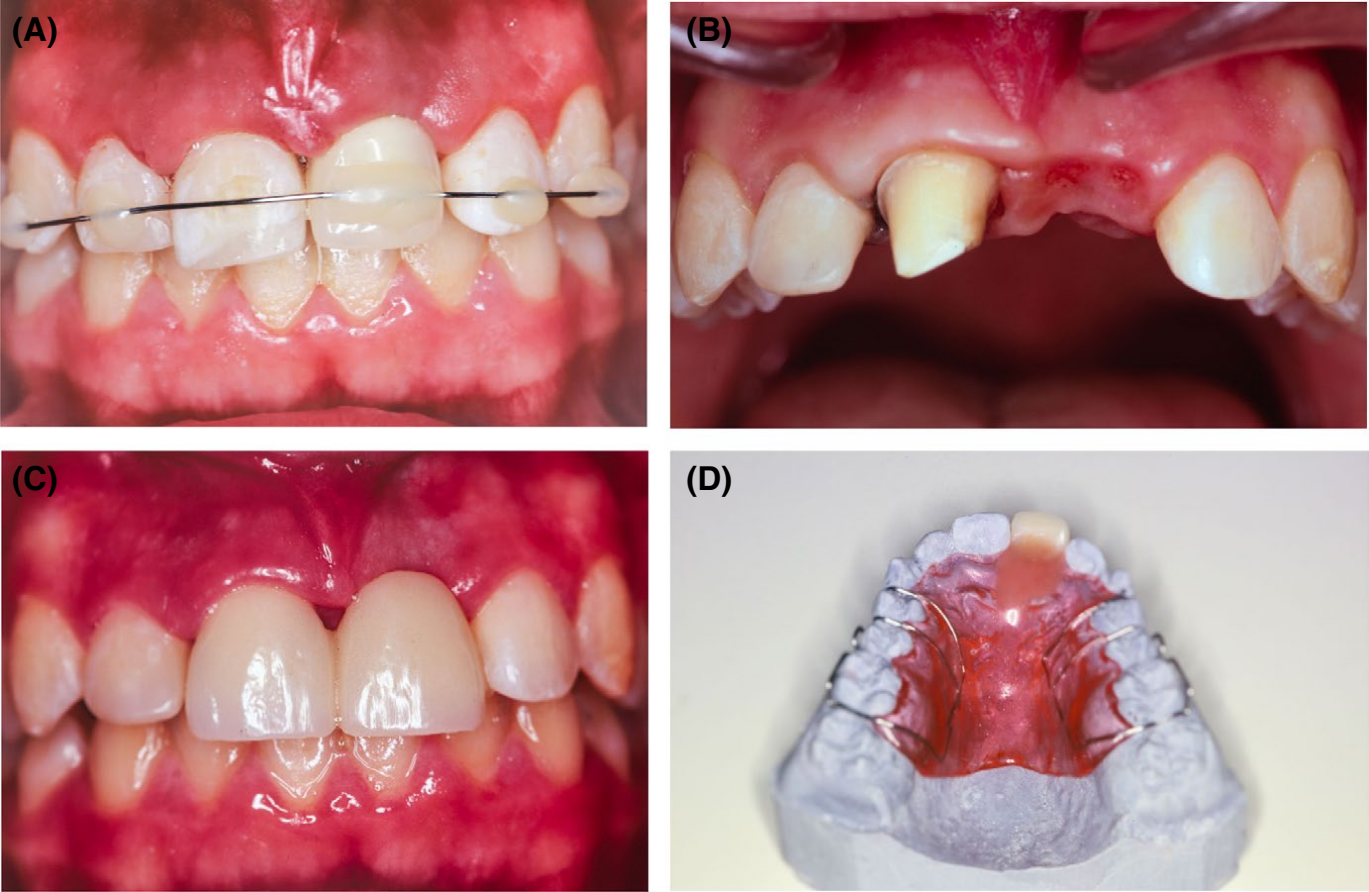
Crown fractures restored with a composite and missing tooth #21 replaced with provisional acrylic tooth 7 days after trauma (A). Tooth #11 was prepared for provisional acrylic crown (B), tooth #21 was firstly replaced with temporary acrylic pontic (#11‐x) prone to fracture (C) and later with removable retainer (D)

**FIGURE 3:**
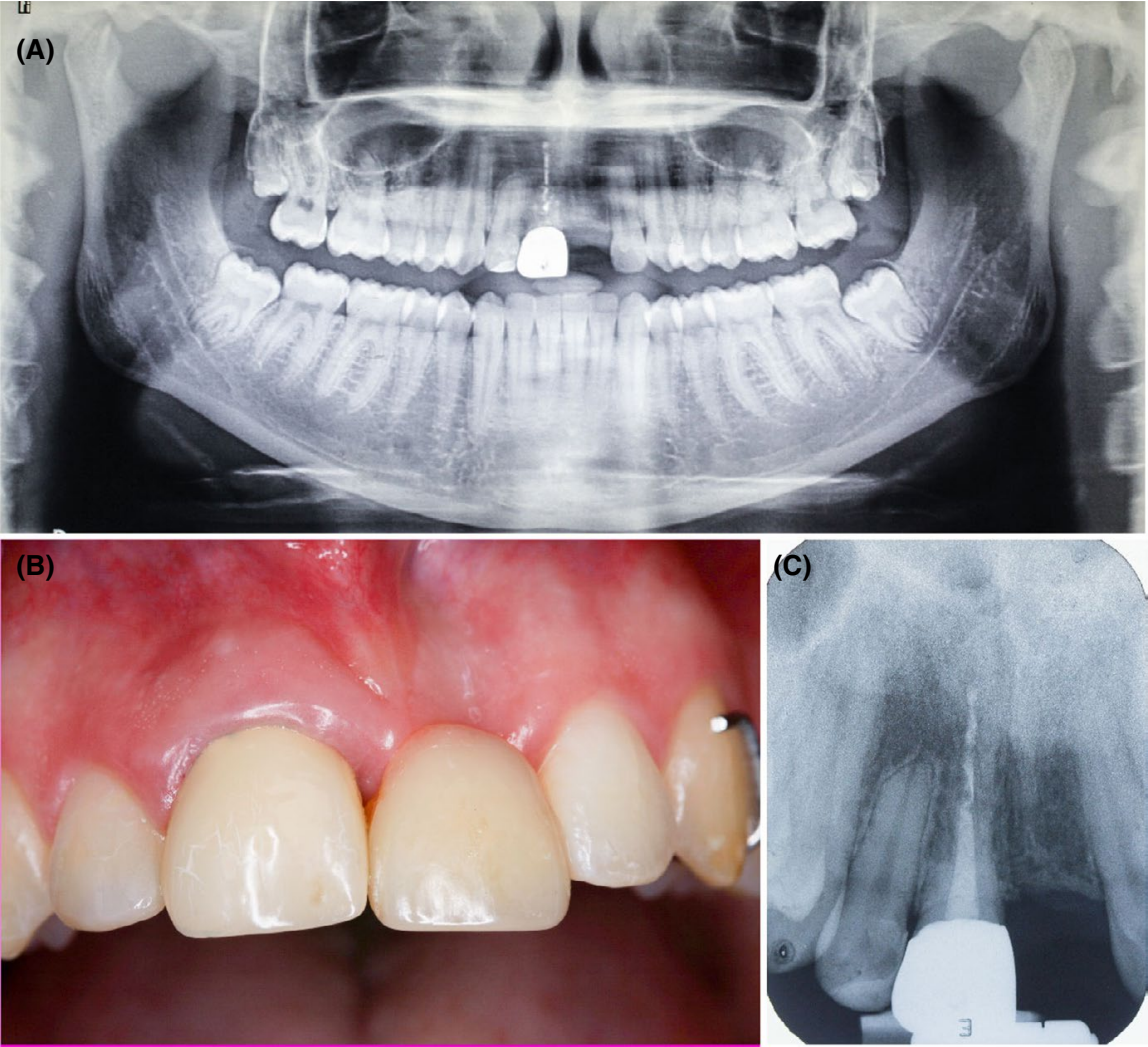
Ortopantomographic image and periapical X‐ray indicating ankylosis of tooth #11 with replacement resorption 2 years after the accident (A, C). Infra‐occlusion of tooth #11 compared to provisional restoration 2 years after trauma (B)

**FIGURE 4:**
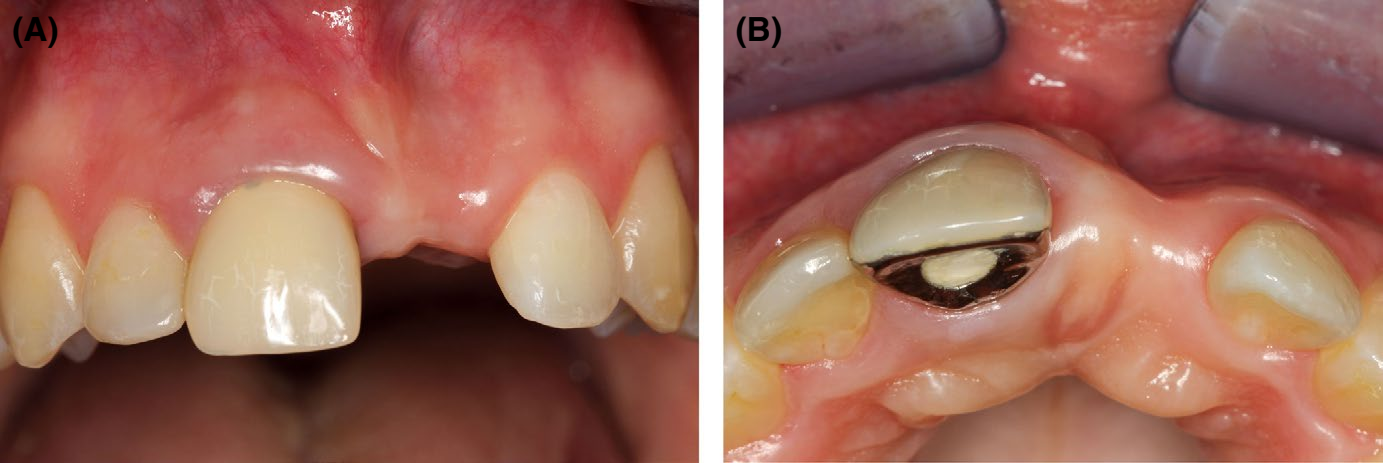
Extensive horizontal bone resorption before the first implant placement 7 years after trauma (A, B)

**FIGURE 5:**
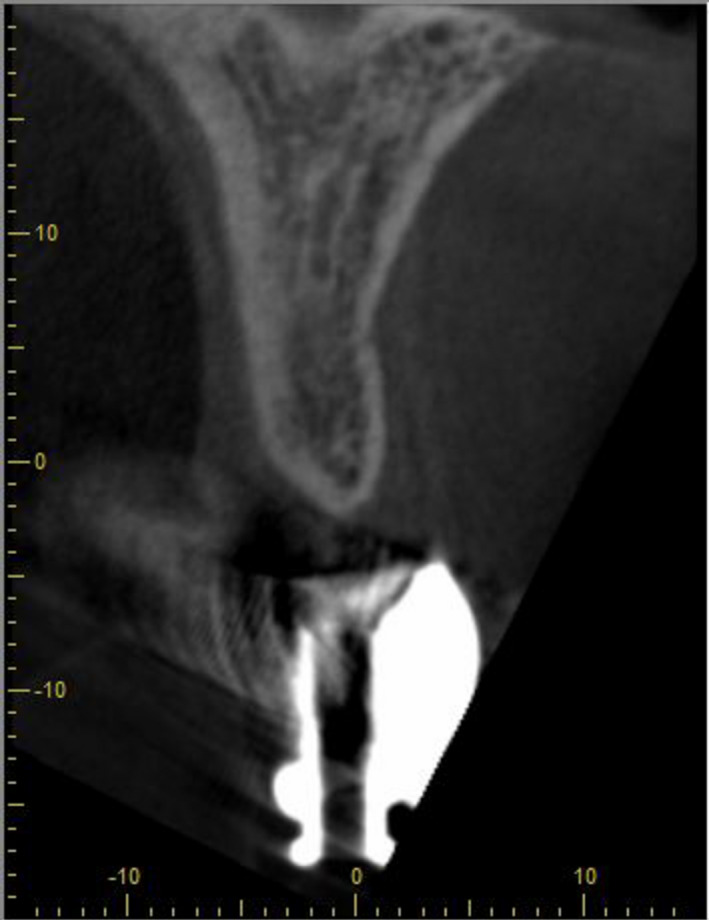
CBCT scan with surgical guide indicating horizontal bone resorption in the region of missing tooth #21 (before implantation)

It was inevitable to postpone implant placement by the end of skeletal growth. Meanwhile, tooth # 11 was treated endodontically with calcium hydroxide paste. Initially, tooth #21 was replaced with provisional acrylic tooth included into a flexible wire splint (Figure 2A). The splint was removed 14 days after the traumatic injury and the missing tooth #21 first replaced by acrylic fixed partial denture prone to fracture; therefore, a removable denture was introduced (Figure 2B–D). Long‐term prognosis of tooth #11 after delayed replantation was considered hopeless. Ankylosis and resorption of the root were expected in the future.^1^, ^2^ Calcium hydroxide was continuously used as intracanal medicament. After 2 years, obvious signs of tooth #11 ankylosis were confirmed (high percussion sound, replacement resorption, slight infraposition)^1^ (Figure 3A–C) and alveolar ridge in the region of #21 underwent extensive horizontal resorption (Figures 4 and 5). At the same time, comprehensive evaluation of tooth #12 demonstrated radiographically visible apical root resorption (Figure 3A,C). In the next 5 years, the patient was regularly re‐examined every 6 months to exclude possible severe consequences of ankylosis (tooth #11 infraposition or tilting of tooth #12).

#### 2.2.2 Surgical and restorative phase

**FIGURE 6:**
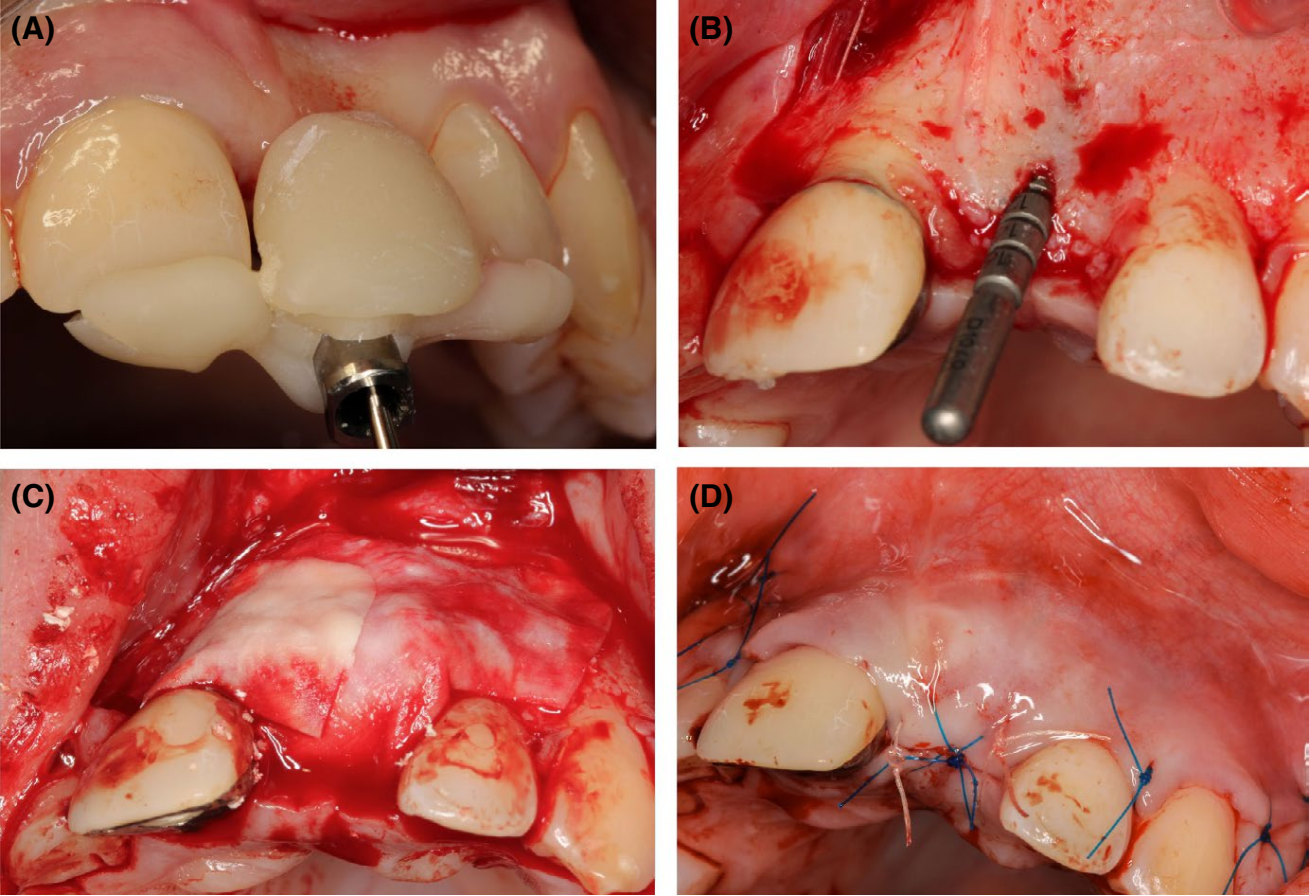
Implant placement #21 with simultaneous GBR 7 years after trauma; surgical guide (A), operating field after trapezoidal mucoperiostal flap elevation and pilot depth gauge (B), double layered technique for membrane stabilization (C), operating field with sutures (D)

**FIGURE 7:**
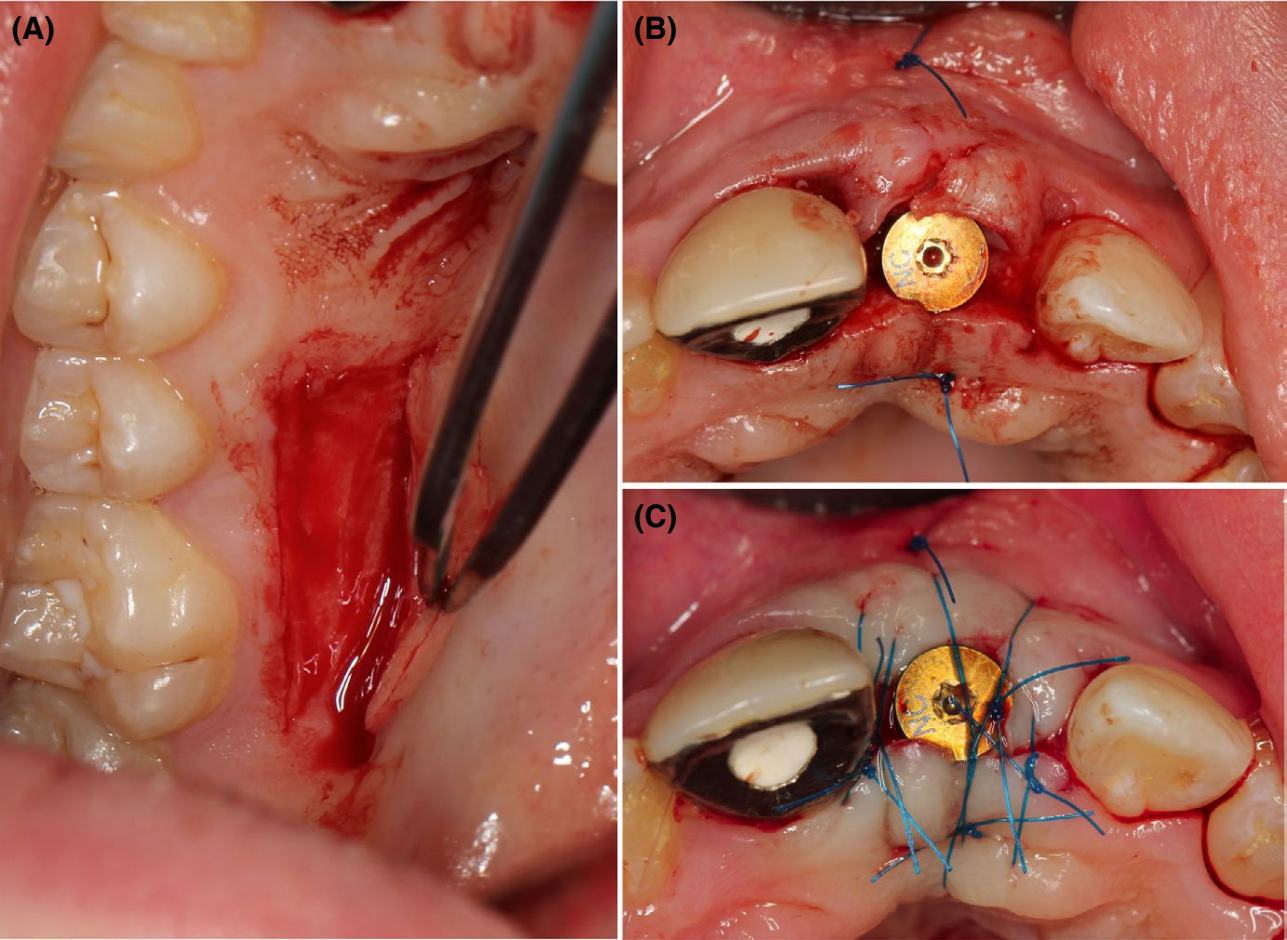
Implant #21: During the second stage after 6 months, subepithelial connective tissue graft harvested by trap‐door technique (A) was transplanted to the region of #21 for contour augmentation (B, C)

**FIGURE 8:**
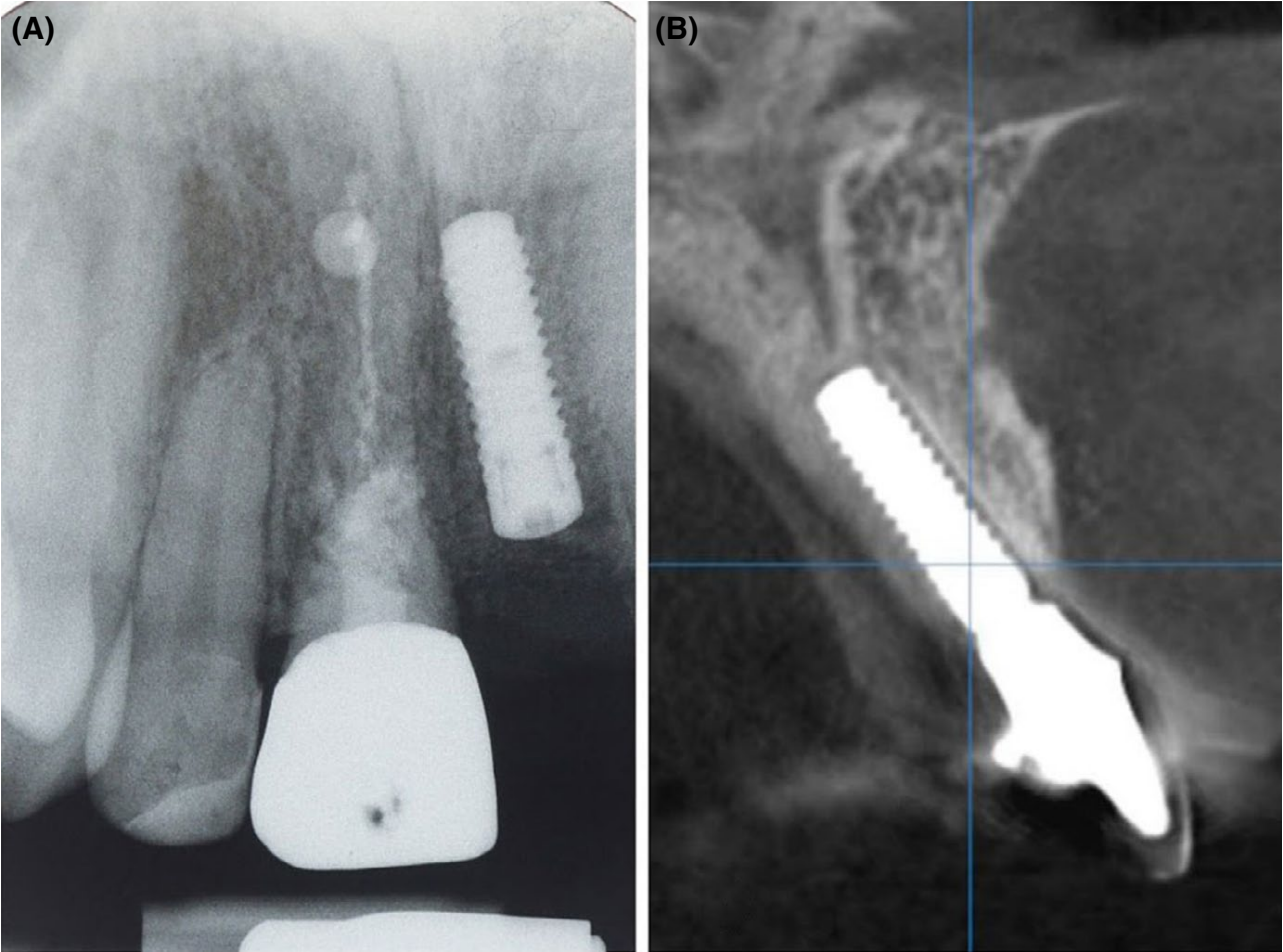
X‐ray image immediately after #21 implant placement (A) and CBCT scan in the region of #21 1 year postoperatively (implant #21 with provisional crown) (B)

**FIGURE 9:**
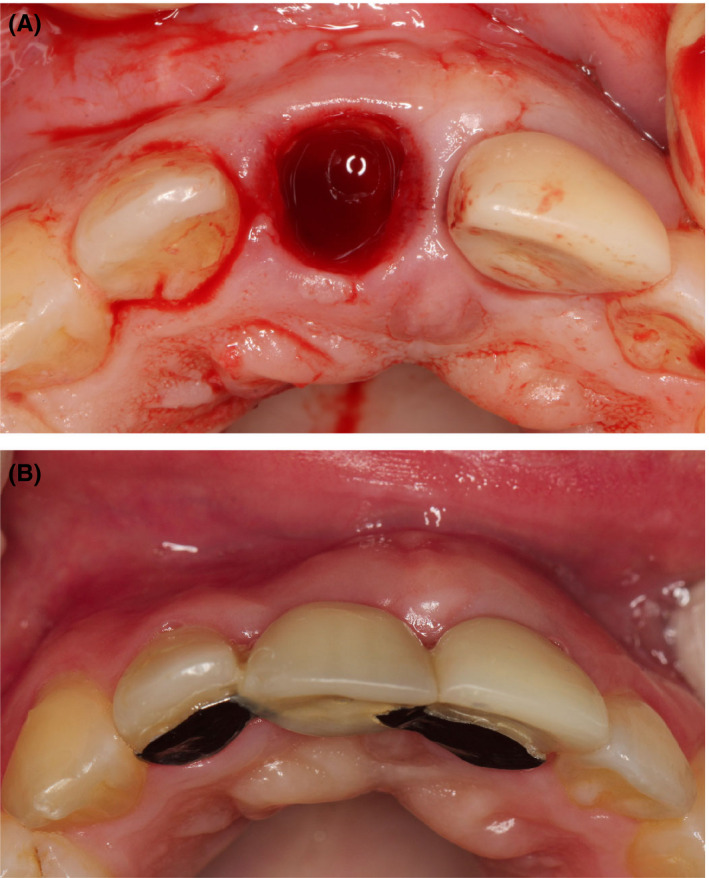
Socket‐shield technique 1 year after provisional restoration of implant #21 (A). Temporary resin‐bonded Maryland bridge for replacement of #11 (B)

**FIGURE 10:**
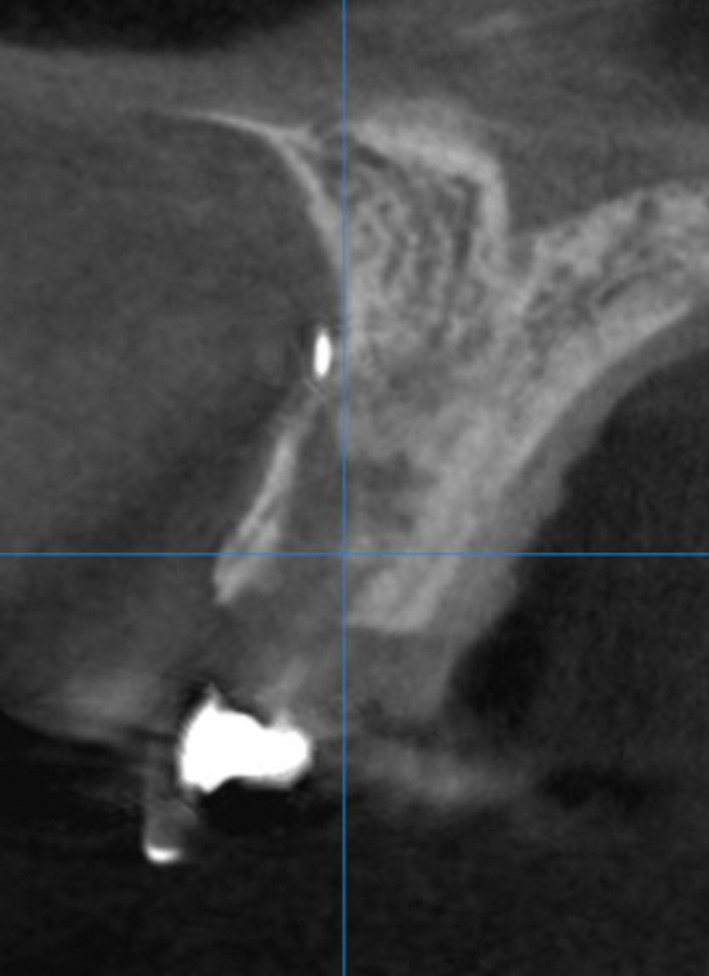
CBCT scan before implant placement into region of #11

**FIGURE 11:**
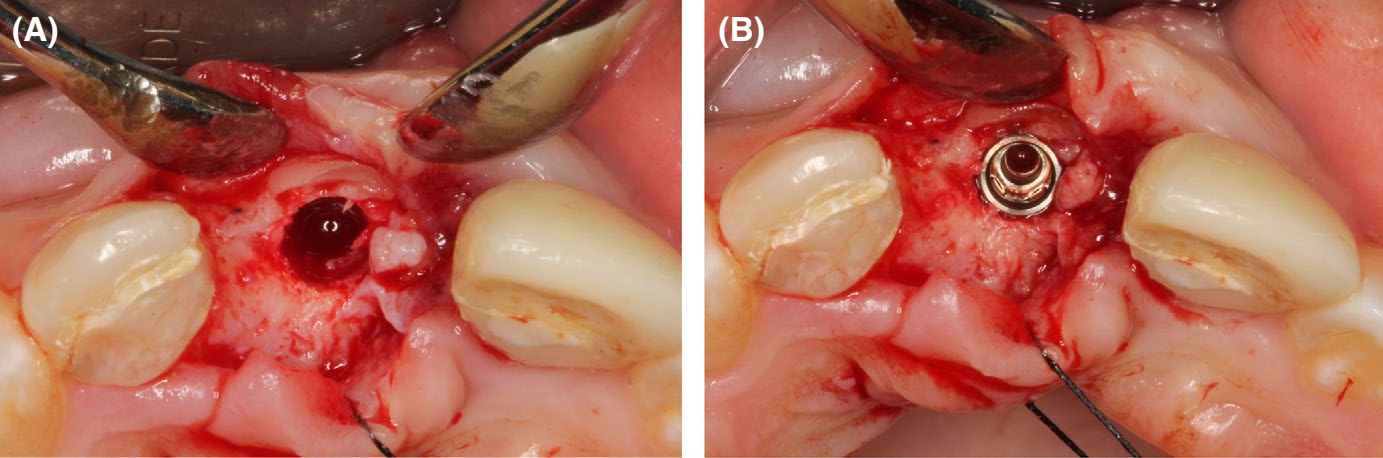
Remains of buccal root lamina (A) and position of implant #11 intra‐operatively 6 months after socket‐shield technique (B)

**FIGURE 12:**
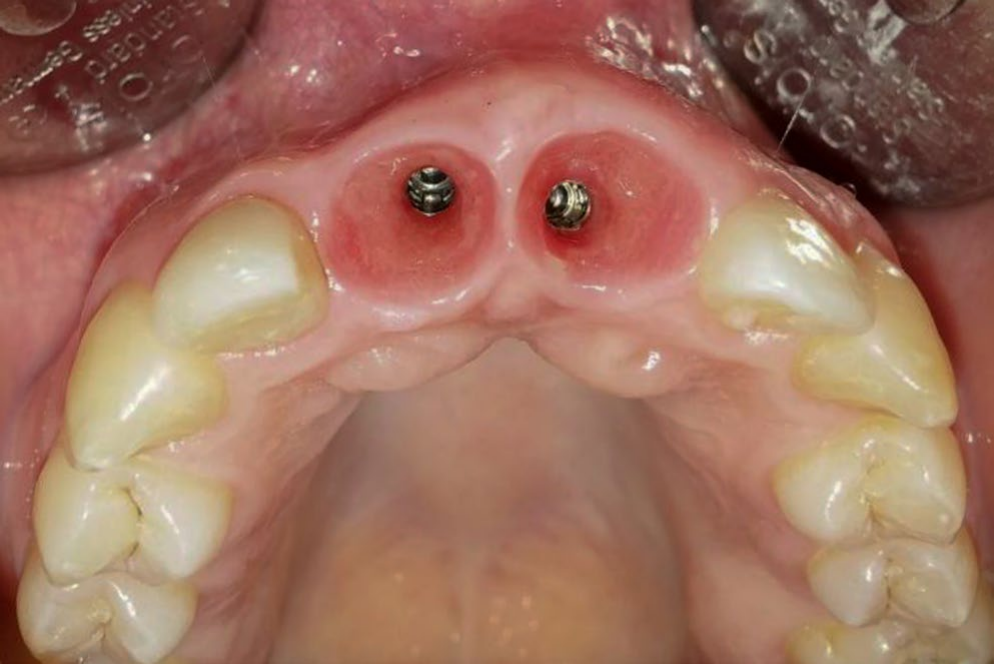
Natural gingival contour including interdental papillae after soft tissue management

Seven years after trauma (at the age of 20), implant (Straumann BL NC Roxolid SLA active 12 mm)(Figure 6A) was placed on position of #21 with simultaneous guided bone regeneration (GBR) using composite bone graft (auto‐ and xenograft) (Geistlich BioOss 0.25–1 mm, Geistlich Pharma AG) and two non‐crosslinked collagen membranes (Jason membrane, Botiss biomaterials GmbH and Bio‐Gide, Geistlich Pharma AG). A double layer technique and fixation with pins to improve membrane stability were utilized (Figures 6B–C and 8A).^7^ Pre‐determined 3D implant position was achieved by using surgical guide for a pilot drill and verified by acrylic mock‐up. After 6 months, subepithelial connective tissue graft harvested by trap‐door technique^8^ was transplanted to the region of #21 for contour augmentation (Figure 7A–C). One year after provisional restoration of implant #21 (Figure 8B), ankylosed tooth #11 was drilled out of the alveolar ridge leaving the buccal root lamina for socket shield (Figure 9A).^9^ Original socket‐shield technique^9^ usually requires immediate implant insertion. Our modification was aiming at the spontaneous bone regeneration of the alveolar ridge palatal to the buccal shield without the use of additional fillers. The second dental implant (Straumann BL NC Roxolid SLA active 12 mm) was therefore inserted 6 months after partial extraction of #11 (Figures 10 and 11A,B) into regenerated bone 1 mm palatal to the buccal shield. Three months later soft tissue management followed. Composite resin was repetitively added to the provisional acrylic crowns at frequent intervals to create a convex cervical shape (Figure 12), ending up with a final aesthetically acceptable rehabilitation with two screw‐retained all‐ceramic crowns.

### 2.3 Clinical outcome and follow‐up

**FIGURE 13:**
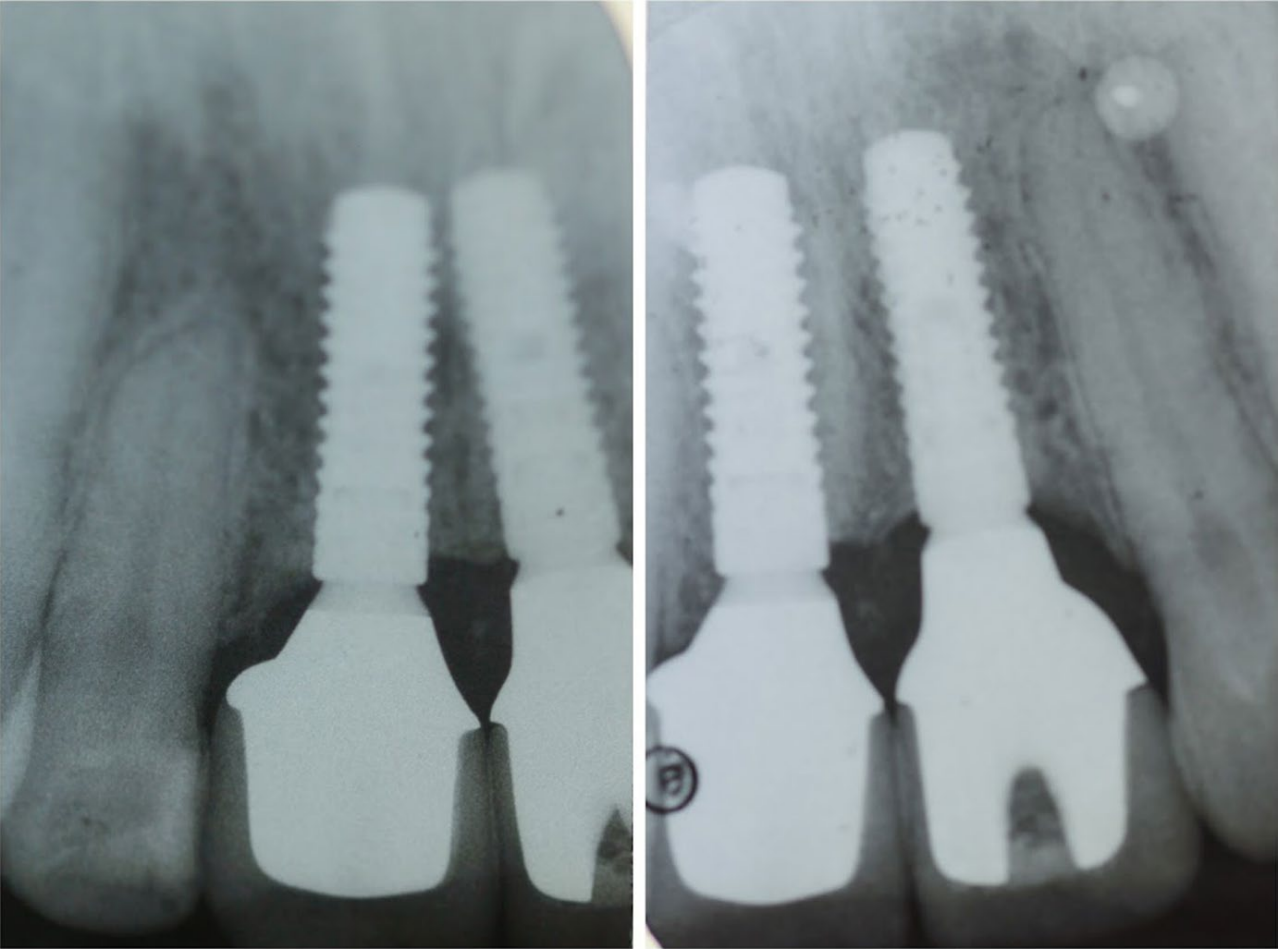
Periapical X‐rays of both implants 3 years after final prosthetic rehabilitation

**FIGURE 14:**
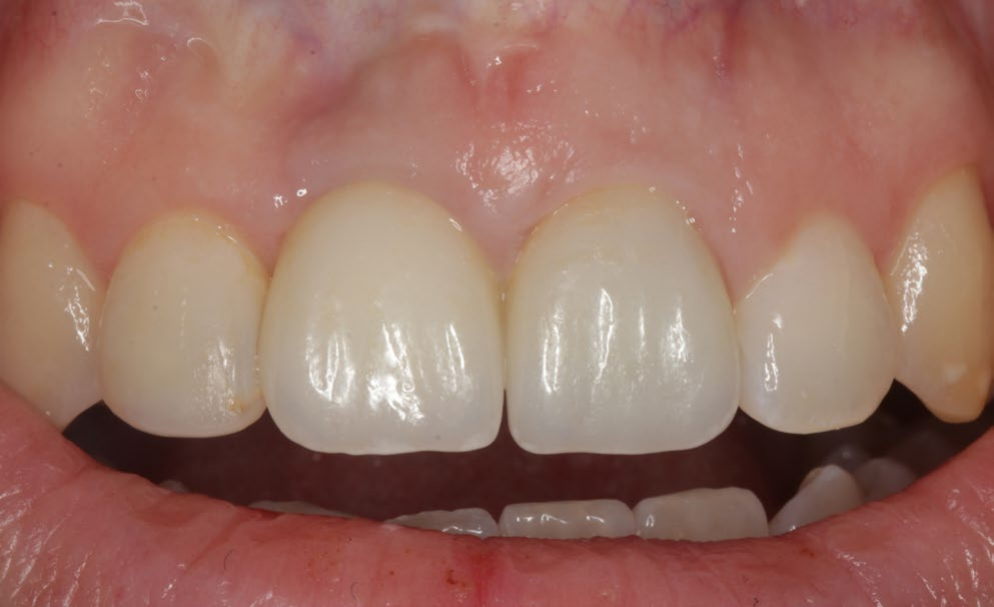
Final aesthetic result of two individual screw‐retained all‐ceramic crowns 3 years after completion—frontal view

**FIGURE 15:**
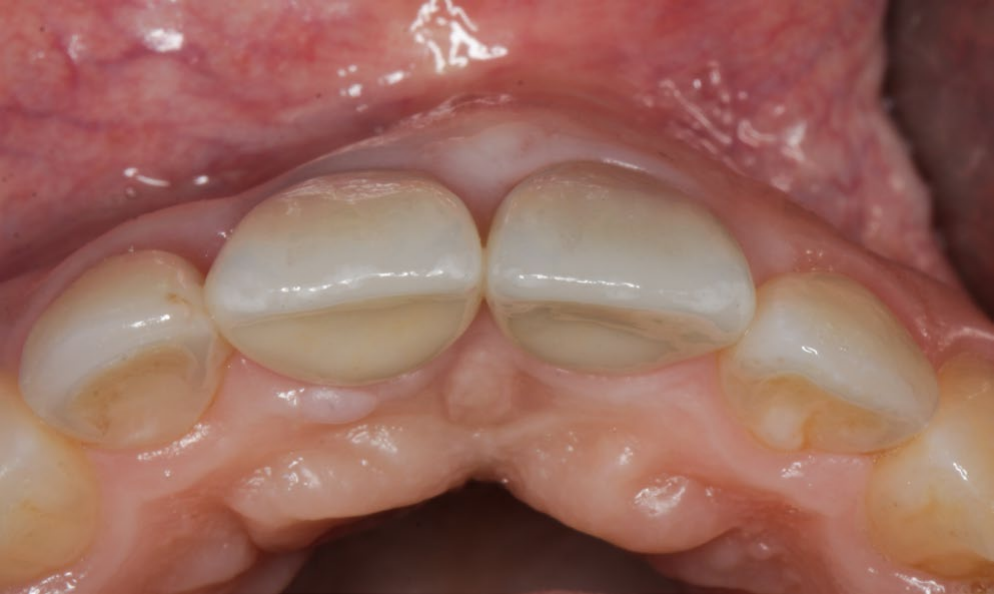
Final aesthetic result of two individual screw‐retained all‐ceramic crowns 3 years after completion—occlusal view

**FIGURE 16:**
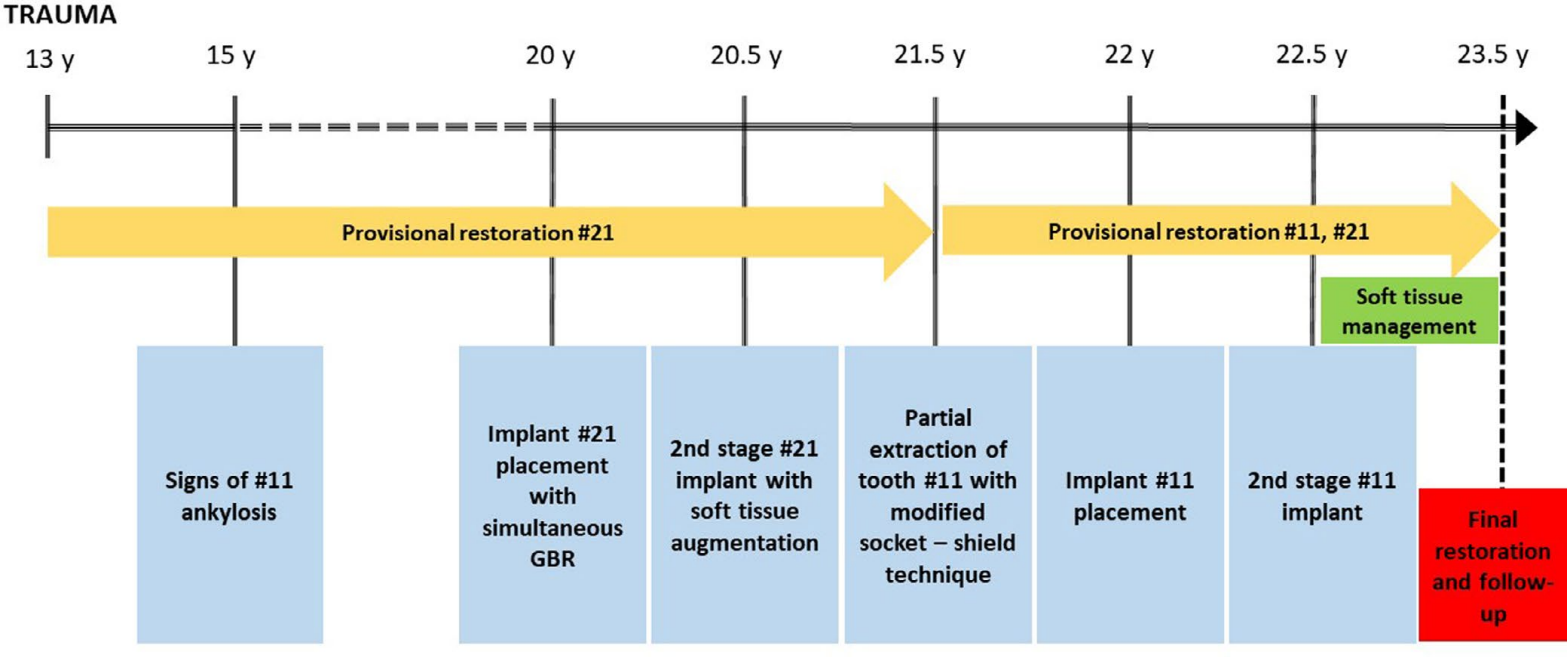
Sequential order of the treatment represented with a flowchart

The primary goals of soft tissue contours re‐creation and preservation of central papillae after a decade‐long treatment was achieved and a pleasant smile was created for the patient. Clinical and radiographic follow‐ups are regularly performed in 6‐month intervals. Mesial and distal papillae complete interdental as well as inter‐implant spaces, there is also no soft tissue level nor texture discrepancy. Due to different reflection of light, the peri‐implant mucosa appears slightly gray. A critical assessment of peri‐implant soft tissue based on the pink aesthetic score (PES)^10^ accentuates differences in soft tissue color and contour with less favorable results at the site of previously ankylosed tooth #11. The area of implant #11 consequently ended with slightly lower, yet aesthetically acceptable aesthetic outcome in the means of color and contour (PES =11) when compared to neighboring teeth and implant #21 with maximal PES (PES =14).^10^ During 3 years of follow‐up, both implant‐supported crowns enable good oral maintenance care and exhibit excellent function. Clinical and radiographic examination of tooth #12 and implants #11, #21 demonstrated the absence of ongoing clinical pathology (Figures 13, 14 and 15). All treatment steps are presented with a flowchart (Figure 16).

## 3 DISCUSSION

Treatment alternatives for replacement of one or more maxillary incisors that are lost or deemed to be lost due to avulsion or late replantation depend on the patient's age at the time of trauma as well as his/her social and aesthetic expectations, anticipated complications (ankylosis, replacement and inflammatory resorption, infraposition, tilting of adjacent teeth) and clinical and technical skills of the appointed dentists. Multiple solutions are available which include autotransplanted permanent teeth, orthodontic space closure, fixed or removable partial dentures, and implant‐borne prosthesis.^11^

Replacement of missing maxillary incisors with a suitable transplant (mainly premolar) is mostly considered optimal when the root development of the transplant has reached two‐thirds to three‐quarters of the expected final root length. Perfect candidates suited for autotransplantation of premolars to the maxillary anterior region are usually about 12 years of age, which corresponds with the period when most serious traumatic dental injuries occur in children.^11^ Our patient was evidently not appropriate for such treatment as she continuously refused any sort of extraction; moreover, the roots of her premolars were fully developed lowering the odds for successful revascularization. Apical root resorption in tooth #12, in addition to optimal occlusion, precluded orthodontic space closure.

When implant‐supported crowns are selected, clinicians must bear in mind the utmost importance of postponing the implant placement by the end of skeletal growth. During the interim period which may last several years, the patient needs to wear either a fixed partial denture or removable appliance prone to fracture. The second challenge arises from the continuous reduction in both osseous and soft tissue support.^5^, ^11^ Another limitation for implant placement in young adults results from the possibility of implant infraposition and aesthetic concerns over time.^12^

Although single tooth replacement in the aesthetic zone is a frequent indication for implant therapy with long‐term success and high patient satisfaction,^3^, ^13^ even in situations with young adult patients,^14^ indications for multiple implant‐supported restorations in the anterior maxilla are rarely described in the literature. Aesthetically disturbing complication of such procedures may result from un‐optimal blend and harmony within existing dentition due to the absence of inter‐implant papillae and buccal soft tissue dehiscences (BSTD).^13^, ^15^ Identified risk factors for BTSD include a thin tissue biotype and a facial malposition of the implant.^3^ Therefore, it is imperative to ensure the correct 3D implant position using surgical guides. Concerning the importance of buccal bone dimensions, its influence on the development of BSTD is currently not well understood.^13^, ^15^

Thus, in our case, a naturally scalloped gingival appearance including interdental papillae putatively resulted from stepwise replacement of both missing central incisors with utilization of surgical guides to facilitate adequate 3D position and temporary preservation of hopeless ankylosed tooth. The patient was very sensitive in the early period of the treatment refusing any procedures involving extraction or decoronation of ankylotic tooth.^11^, ^16^, ^17^ At the age of 20 when the alveolar ridge was considered adequately developed, the first implant was inserted with simultaneous guided bone regeneration (GBR) procedure. In simultaneous implant placement and GBR, a resorbable collagen membranes and a mixture of particulate xenogenic bone particles with autologous bone yields stable and predictive long‐term results for single‐site horizontal ridge augmentation..^3^, ^7^, ^18^ At the opening, buccal contour of the site was additionally augmented with connective tissue graft.^19^ Ankylotic tooth was kept and drilled out with buccal root fragment left for socket preservation. In our modification of socket‐shield technique,^20^ partial extraction with residual root fragment located in bucco‐coronal part of the socket enabled spontaneous bone regeneration and socket preservation without any filler material. The other option would have included implant placement with simultaneous GBR. Yet, such technique would have required extensive mucoperiostal flap elevation with possible damage to central papilla. The area of implant #11 consequently ended with aesthetically acceptable outcome that was in terms of PES slightly lower in comparison with site treated with GBR and connective tissue graft. It should be noted that similar acceptable PES values were reported for majority of single implants placed in aesthetic region according to delayed implantation protocol.^13^ Nevertheless, soft tissue augmentation with connective tissue graft to further improve buccal contour in the slightly underdeveloped region of formerly ankylosed tooth #11 is planned for the future.^19^

Besides correct 3D implant positioning together with adequate quantity and quality of peri‐implant tissues after surgical procedures, a satisfying emergence profile and gingival architecture was finally achieved by repetitive composite reshaping of the temporary restorations. Such prosthetic management was crucial in maximizing the aesthetic result. Yet, application of pressure to the marginal gingiva must be performed with extreme caution to evade pain, anemia, or tissue necrosis.^21^

## 4 CONCLUSION

To the best of our knowledge, this is the first case report on stepwise replacement of traumatized central maxillary incisors for two single implant‐supported crowns with temporary preservation of ankylosed tooth. It may represent a rare clinical indication in cases where one incisor is lost and the other ankylotic incisor does not cause severe infraposition or tilting of adjacent teeth in a patient who has passed pubertal growth spurt. Such solution requires interdisciplinary approach, with special efforts put into proper diagnosis and careful execution of all required clinical procedures to yield functional and aesthetically acceptable result.

## AUTHOR CONTRIBUTIONS

RG and RK planned the stepwise treatment approach, collected the clinical data and images. RK participated in initial examination and diagnosis; was in charge of conservative phase. RG took the lead in surgical part of the treatment; has been performing follow‐up care. ACK took the lead in material preparation and writing the first and final draft. KVG was involved in prosthodontic treatment of the patient. All authors reviewed and approved the final manuscript.

## CONSENT

Submitted and published with written consent of the patient.

## Data Availability

The data that support the findings of this case report are available from the corresponding author upon reasonable request.
